# Third ventricular width by transcranial sonography is associated with cognitive impairment in Parkinson's disease

**DOI:** 10.1111/cns.14360

**Published:** 2023-07-13

**Authors:** Hong‐ling Gao, Yi Qu, Sheng‐chong Chen, Qing‐mei Yang, Jing‐yi Li, An‐yu Tao, Zhi‐juan Mao, Zheng Xue

**Affiliations:** ^1^ Department of Neurology, Tongji Hospital, Tongji Medical College Huazhong University of Science and Technology Wuhan China; ^2^ Division of Pulmonary and Critical Care Medicine, Department of Internal Medicine, Tongji Hospital, Tongji Medical College Huazhong University of Science and Technology Wuhan China; ^3^ Department of Ultrasound, Tongji Hospital, Tongji Medical College Huazhong University of Science and Technology Wuhan China

**Keywords:** cognition, Doppler, Parkinson's disease, third ventricular width, transcranial sonography

## Abstract

**Background:**

One‐fourth of Parkinson's disease (PD) patients suffer from cognitive impairment. However, few neuroimaging markers have been identified regarding cognitive impairment in PD.

**Objective:**

This study aimed to explore the association between third ventricular width by transcranial sonography (TCS) and cognitive decline in PD.

**Method:**

Participants with PD were recruited from one medical center in China. Third ventricular width was assessed by TCS, and cognitive function was analyzed by the Mini‐Mental State Examination (MMSE). Receiver operating characteristic (ROC) analysis and Cox model analysis were utilized to determine the diagnostic and predictive accuracy of third ventricular width by TCS for cognitive decline in PD patients.

**Result:**

A total of 174 PD patients were recruited. Third ventricular width was negatively correlated with MMSE scores. ROC analysis suggested that the optimal cutoff point for third ventricular width in screening for cognitive impairment in PD was 4.75 mm (sensitivity 62.7%; specificity 75.6%). After 21.5 (18.0, 26.0) months of follow‐up in PD patients without cognitive impairment, it was found that those with a third ventricular width greater than 4.75 mm exhibited a 7.975 times higher risk of developing cognitive impairment [hazard ratio = 7.975, 95% CI 1.609, 39.532, *p* = 0.011] compared with patients with a third ventricular width less than 4.75 mm.

**Conclusion:**

Third ventricular width based on TCS emerged as an independent predictor of developing cognitive impairment in PD patients.

## Graphical abstract

A total of 174 PD patients were recruited, and all of them completed TCS examination and Mini‐Mental State Examination (MMSE) assessment. At the last, our findings suggested that larger third ventricular width and poor cognition. Furthermore, ROC analysis suggested that the optimal cutoff point for third ventricular width in screening for cognitive impairment in PD was 4.75 mm (sensitivity 62.7%; specificity 75.6%). After 21.5 (18.0, 26.0) months of follow‐up in PD patients without cognitive impairment, it was found that those with a third ventricular width greater than 4.75 mm exhibited a 7.975 times higher risk of developing cognitive impairment compared to patients with a third ventricular width less than 4.75 mm.

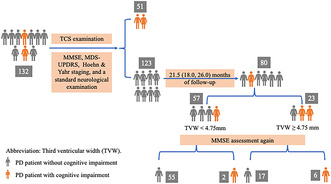

## 1 INTRODUCTION

Cognitive impairment is one of the most devastating nonmotor symptoms, occurring in approximately 25%–30% of Parkinson's disease (PD) patients.^1^ The actual number of PD patients with cognitive dysfunction may exceed the statistics of clinical diagnosis.^2^ Cognitive dysfunction seriously interferes with motor function and quality of daily life,^3^ shortens patient survival time and increases mortality,^4^ and imposes substantial burdens on families and public health care.^3^ In 2020, the Lancet Committee indicated that nearly 50% of dementia patients could have been prevented or delayed by controlling risk factors.^5^ Therefore, the search for markers to predict and identify cognitive decline in PD patients early is very important.

A review^1^ has shown that brain volume atrophy and widening of the sulci and reduction in the gyri in the hippocampus are the pathological manifestations of PD patients with cognitive impairment. Progressive brain atrophy in PD patients has been identified as a structural imaging marker of cognitive decline.^6^ Notably, two‐dimensional measures of ventricular enlargement are reproducible and clinically relevant markers of brain atrophy.^7^ A study has found significant correlations between the third ventricle width as seen on brain magnetic resonance images (MRI) and cognitive impairment in both Alzheimer's disease (AD) and Lewy body dementia (LBD).^8^ In healthy individuals, another study has suggested that third ventricular width in transcranial sonography (TCS) is indicative of brain atrophy associated with physiological aging and dementia.^9^ A cross‐sectional study has further demonstrated an association between third ventricular width and cognitive decline in PD patients.^10^ Differences in brain structural parameters are well recognized among different racial and ethnic groups. For example, the optimal cutoff points for TCS in diagnosing PD vary between different races.^11^, ^12^, ^13^ However, the correlation between third ventricular width and cognitive impairment in PD patients in China has yet to be established.

The goal of this study was to explore the association between third ventricular width and cognitive dysfunction in PD patients in China and to identify diagnostic tools capable of capturing the cognitive changes occurring throughout PD.

## 2 PATIENTS and METHODS

### 2.1 Participants and clinical scales

A total of 174 PD patients followed up at Tongji Hospital Affiliated to Tongji Medical College of Huazhong University of Science and Technology were included in this study from November 2017 to October 2020. All participants had a diagnosis of PD according to Movement Disorder Society (MDS) criteria.^14^ They underwent detailed history taking and MRI to ensure that they had no cognitive impairment or diseases affecting cognitive function before the onset of PD. Exclusion criteria included (i) severe cardiovascular, dementia, corticobasal degeneration, tumors, trauma, infarction, hemorrhage, or extensive white matter lesions; (ii) previous medical history of deep brain stimulation (DBS), pallidotomy, and stem cell therapy; (iii) previous manifestations of cognitive impairment and diseases that may affect cognitive function; and (iv) cognitive impairment occurring before or within 1 year of the onset of PD. Furthermore, patients with an insufficient temporal bone window were not included in the calculation and statistical analysis.

In addition, they also completed a thorough set of clinical examinations, which included Hoehn & Yahr staging, the Movement Disorder Society Unified Parkinson's Disease Rating Scale (MDS‐UPDRS, Part I = Non‐Motor Aspects of Experiences of Daily Living, Part II = Motor Aspects of Experiences of Daily Living, Part III = Motor Examination), and the Mini‐Mental State Examination (MMSE), a standard neurological examination, and TCS examination.

The study was approved by the ethics committee of Tongji Hospital affiliated with Tongji Medical College of Huazhong University of Science and Technology (IRB 2020S108). All participants provided informed consent before enrollment.

### 2.2 Cognitive assessment

The MMSE is widely and most commonly used to evaluate cognition in clinical practice.^15^ The MMSE consists of five dimensions: visuospatial and executive functioning, language abilities, short‐term memory/delayed recall, attention, and orientation. The score for each dimension is summed to obtain the overall MMSE score, which ranges from 0 to 30, with higher scores indicating better cognitive performance. MMSE scores were categorized into not cognitively impaired and cognitively impaired. Based on the Chinese Revised MMSE scoring standard,^16^ the participants with illiteracy scoring ≤17 points, those with primary school education scoring ≤20 points, and those with secondary school or above education scoring ≤24 points were included in the PD with cognitive impairment group, and others were included in the PD without cognitive impairment group. The PD with cognitive impairment group included 51 patients, and the other group included 123 patients. At follow‐up, of those without cognitive impairment, two patients died, 10 patients were lost to follow‐up, and 31 patients were not included because the follow‐up time was less than 1 year. The follow‐up analytic population included 80 patients who completed the MMSE to assess cognitive function again. Among all follow‐up subjects, eight patients with PD developed cognitive impairment.

### 2.3 Transcranial sonography

**FIGURE 1:**
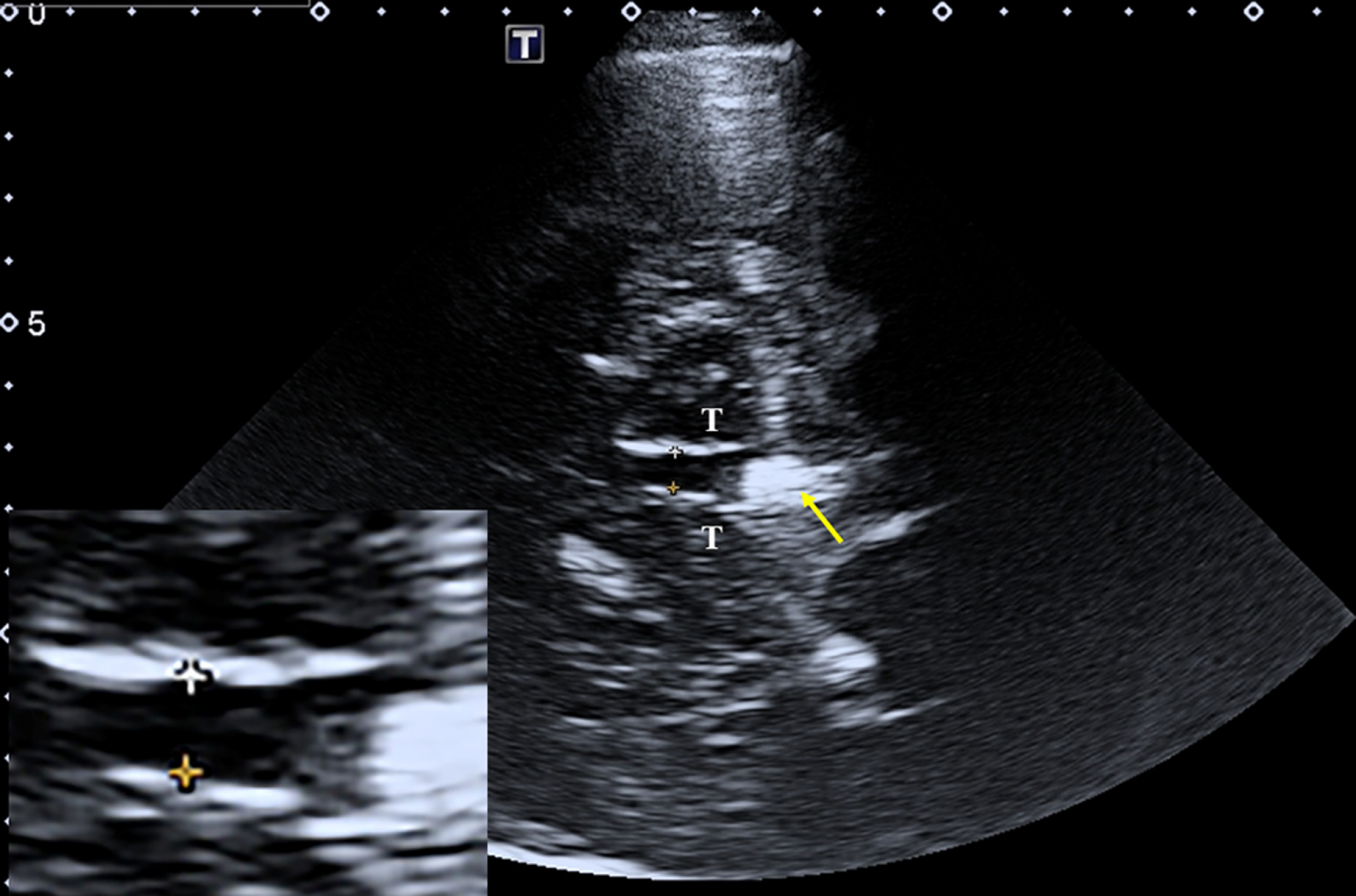
TCS images of the scanning planes at the level of the thalamus in a PD patient. TCS image of axial section at the level of the thalamus. The yellow arrow indicates the pineal gland that can be regularly observed on TCS as a “sonographic landmark” of high echogenicity owing to calcification. T, thalamus; TCS, transcranial sonography. 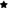
Measuring points for three ventricular widths.

To ensure the reproducibility and reliability of measurements, TCS examinations for each participant were conducted independently by two sonographers who were blinded to the other imaging results of the patients. TCS images were stored digitally, patient names were deleted, and the images were labeled with new computer‐generated numbers by a clinical worker. The two sonographers were blinded to the randomized numbers, read the TCS images, and measured related parameters a month later. If uncertainties about TCS imaging arose, each sonographer consulted with a third sonographer independently. The ultrasound system was a color‐coded, phased‐array ultrasound system equipped with a 2.0–3.0 MHz transducer (Toshiba APL500). TCS settings and parameters included a penetration depth of 14–16 cm and a dynamic range of 45–55 dB when sonographers explored echogenicity in the midbrain through the temporal bone. Starting from the mesencephalic plane, the ultrasound section through the thalamus was displayed by tilting the ultrasound beam approximately 10°–20° upward (Figure 1). At this plane, the transverse diameter of the third ventricle and of the frontal horn of the contralateral lateral ventricle can be measured.^17^ For accurate and reproducible ventricular width measurements, TCS measurements were performed from the ipsilateral to the contralateral inner layer of the hyperechogenic ependyma as previously described.^18^ In our study, a total of 48 participants were excluded due to the poor quality of the unilateral or bilateral temporal acoustic bone window, which suggested that 21.6% of participants did not have sufficient bone windows to complete the TCS examination.

### 2.4 Statistics

The discrete variables are expressed in absolute values and percentages, while the continuous variables are represented by medians and interquartile ranges [M(IQR)]. Third ventricular width was analyzed as a continuous variable after normalizing, while MMSE was analyzed as both a continuous and binary (cognitively impaired or not cognitively impaired) outcome. The characteristics of participants were compared between with and without cognitive impairment, which applied unpaired independent *t*‐tests for continuous variables with normal distributions, nonparametric Mann–Whitney *U*‐tests for continuous variables without normal distributions, and chi‐squared tests for discrete variables. Spearman correlation analysis and partial correlation analysis were performed to explore the correlations between MMSE scores (continuous) and third ventricular width (continuous). A multiple stepwise linear regression model was applied to estimate the *β* coefficient and 95% confidence interval (95% CI) for associations between the third ventricular width and MMSE score (continuous) with MMSE scores as dependent variables. The covariables included age, onset age, third ventricular width, H–Y stages, and MDS‐UPDRS I, II, and III scores. Binary logistic regression analysis was performed to estimate the odds ratio (OR) and 95% CI for the associations between the third ventricular width and cognitive impairment. Receiver operating characteristic (ROC) curve analysis was used to determine the optimal cutoff points for third ventricular width in diagnosing PD with cognitive impairment. When further analyzing the follow‐up data, we used Cox regression to explore the association between third ventricular width (categorized by the optimal cutoff of third ventricular width by ROC) and cognitive impairment. SPSS 24.0 was used for statistical analysis. Statistical significance was defined as *p* < 0.05. All statistical analysis charts were created by GraphPad Prism 8.

## 3 RESULTS

### 3.1 Demographics and clinical data

A total of 174 patients with PD were included in the study analysis. The baseline demographic and clinical data of the participants can be found in Table S1. The participants had a mean age of 60.00 (51.00, 66.00) years, with 32.2% being women. The mean third ventricular width was 4.40 mm (3.20, 5.60). The mean MMSE score was 26.00 (23.00, 28.00). Based on the H–Y stage classification standard, there were 111 (63.8%) patients with early PD (grade I–II) and 63 (36.2%) patients with advanced PD (grade III–V). There was a significant gender difference in third ventricular width, with the median third ventricular width was greater in men (4.60 mm [3.50, 6.20]) than in women (3.80 mm [2.80, 4.60]) (*p* = 0.001). However, education level and MMSE scores did not differ significantly between genders (*p* > 0.05) (Table S2).

As shown in Table 1, a total of 51 (29.3%) participants had cognitive impairment based on education‐specific cutoffs. Compared with participants without cognitive impairment, participants with cognitive impairment were older [64.0 (58.0, 70.0) vs. 57.0 (50.0, 65.0)] (*p* < 0.001). Although older age and onset age were associated with both lower MMSE scores (Table S3) and larger third ventricular width (Table S4), there was no significant interaction between both variables, and third ventricular width remained independently associated with incident cognitive impairment after adjustment for age and age onset [5.2 (4.2, 7.1) vs. 3.9 (2.9, 4.7)] (*p* < 0.001).

**TABLE 1:** Comparison of disease characteristics between PD patients with and without cognitive impairment.

	PDNC group *n* = 123	PDD group *n* = 51	*p*
Female *n* (%)	41.0 (33.3)	15.0 (29.4)	0.722
Age (years)	57.0 (50.0, 65.0)	64.0 (58.0, 70.0)	<0.001*
Onset of age (years)	52.0 (45.0, 60.0)	58.0 (51.5, 64.5)	0.003*
Duration (years)	4.0 (2.0, 6.0)	4.0 (2.5, 7.0)	0.519
Third ventricular width (mm)	3.9 (2.9, 4.7)	5.2 (4.2, 7.1)	<0.001^a^
H–Y stage	2.0 (1.0, 2.5)	2.0 (1.0, 3.0)	0.840
MDS‐UPDRS score			
Part I	8.0 (5.0, 11.0)	9.0 (6.0, 15.0)	0.116
Part II	8.0 (3.0, 13.0)	8.0 (5.0, 15.0)	0.260
Part III	27.0 (16.0, 41.0)	23.0 (15.0, 42.0)	0.312

Abbreviations: MDS‐UPDRS, Movement Disorder Society Unified Parkinson's Disease Rating Scale; MMSE, Mini‐Mental State Examination; PD, Parkinson's disease, PDD group and PDNC, PD with cognitive impairment group and PD without cognitive impairment group based on MMSE score, respectively.

^a^ *p* value after adjusting age and onset age by binary logistic regression analysis.

* *p* < 0.05, which was statistically significant.

### 3.2 Associations between third ventricular width and cognitive impairment

In the patients with PD, there was a positive correlation between third ventricular width and age (*p* < 0.05) and onset age (*p* < 0.05). Thus, age and onset age adjustments were performed in subsequent analyses to minimize their effects. The partial correlation analysis showed that the associations between third ventricular width and the MMSE scores and memory domain remained significant after adjustments for age and onset age (*p* < 0.001). Detailed information on third ventricular width is presented in Table S4. MMSE scores were negatively associated with age, age of onset, MDS‐UPDRS I score, and third ventricular width (Table S3). A multiple stepwise linear regression analysis was subsequently performed with MMSE scores (continuous) and covariables including age, onset age, gender, education level (categorical variable), H–Y stages, and MDS‐UPDRS I, II, and III scores. The regression model was statistically significant (*p* < 0.001). As demonstrated in Table 2, in the MMSE dependent variable model, the results showed that education level (*p* < 0.001) and third ventricular width (*p* < 0.001) were independent factors influencing cognitive function in PD patients, which explained 23.4% of the variation. Each standard deviation (SD) increment in the third ventricular width score was associated with an increase in mean MMSE score by −0.313 (95% CI −0.799, −0.322).

**TABLE 2:** Associations of third ventricular width with MMSE score and cognitive impairment.

	MMSE score		Cognitive impairment	
	*β* (95% CI)	*p*	OR (95% CI)	*p*
Education level	0.367 (1.200, 2.572)	<0.001*		
Third ventricular width	−0.313 (−0.799, −0.322)	<0.001*	1.631(1.328, 2.004)	<0.001*

Abbreviation: 95% CI, confidence intervals; MMSE, Mini‐Mental State Examination.

* *p* < 0.05, which was statistically significant.

When MMSE was dichotomized into cognitively impaired or not, we performed binary logistic regression with cognitive impairment as the independent variable, and covariables including age, onset age, gender, education level (categorical variable), H–Y stages, and MDS‐UPDRS I, II and III scores. The binary logistic regression confirmed the fitness of our model, which was corroborated by the significance in both Omnibus tests of model coefficients (*p* < 0.001) and the Hosmer and Lemeshow test (*p* = 0.464). Consequently, it was then reasonable to assume that the model would adequately describe the data, and the third ventricular width was responsible for a 22.6% change in the risk of cognitive impairment. Patients with larger third ventricular width were more likely to have cognitive impairment (Table 2). Each SD increment in the third ventricular width was associated with a 63.1% increase in the odds of cognitive impairment (OR 1.631, 95% CI 1.328, 2.004).

### 3.3 ROC analysis of third ventricular width in PD patients with/without cognitive impairment

**FIGURE 2:**
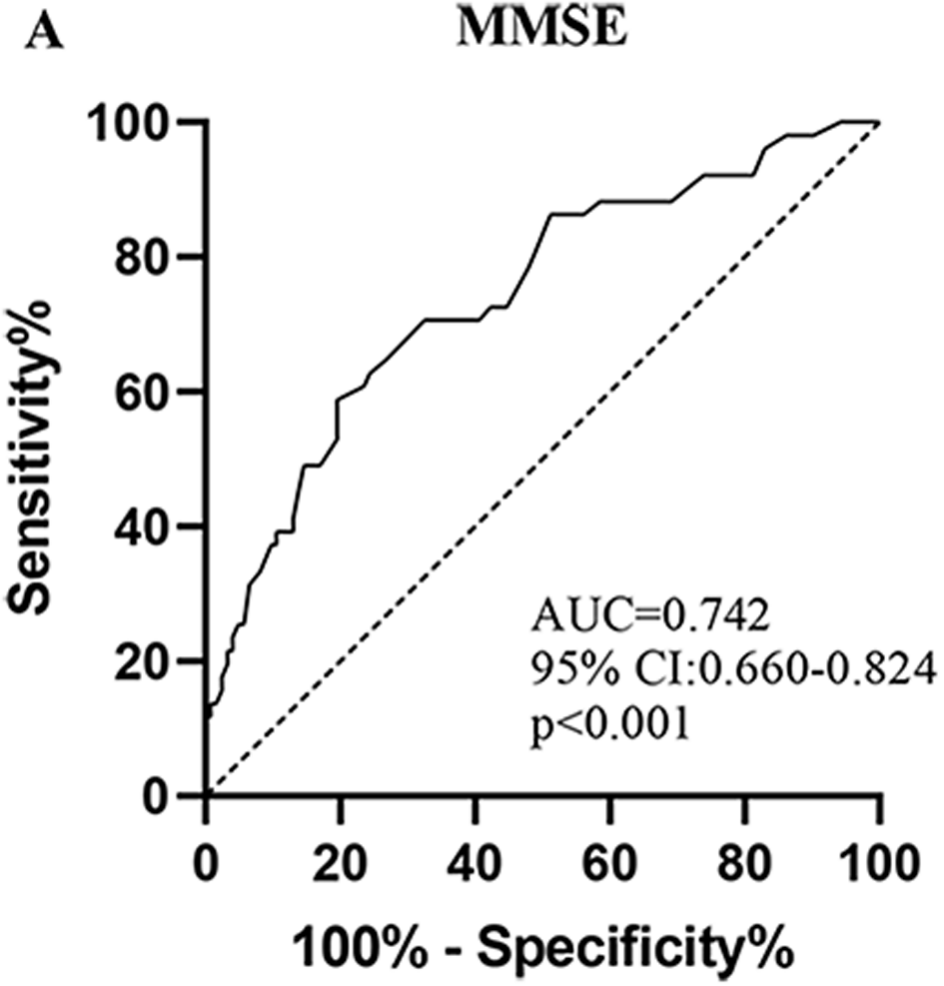
ROC curve analysis for third ventricular width in diagnosing cognitive impairment in PD. MMSE, Mini‐Mental State Examination; PD, Parkinson's disease; ROC, receiver operating characteristic.

A ROC curve analysis was used to determine the accurate diagnosis of third ventricular width regarding cognitive impairment in PD patients and revealed an area under the curve (AUC) of 0.742 (95% CI 0.660, 0.842). This suggested that the third ventricular width could be used as a diagnostic tool in PD with cognitive impairment. The sensitivity was 62.7% for a cutoff value of 4.75 mm, and the specificity was approximately 75.6% (see Figure 2). Thus, patients with a third ventricular width greater than 4.75 mm were more likely to demonstrate cognitive impairment.

### 3.4 Follow‐up analysis of third ventricular width as a predictor of cognitive impairment

In the follow‐up study, the analytic population included 80 patients, of which 26 (32.5%) were women. The median age was 60.0 years (50.0, 66.8), the median age of onset was 55.0 years (46.8, 61.0), the median course of disease was 4.5 years (3.0, 7.0), and the follow‐up time was 21.5 months (18.0, 26.0). Among all follow‐up subjects, eight (10%) PD patients developed cognitive impairment, including three women. The median age for these patients was 65.00 (62.50, 68.00) years, the median age of onset was 56.50 years (55.00, 64.00), the median course of disease was 6.00 years (2.63, 7.00), and the median third ventricular width was 5.65 mm (4.68, 6.45).

To investigate whether the third ventricular width (categorical variable) was the major factor, we included sex, age, age of onset, and third ventricular width (<4.75 mm or ≥4.75 mm) as independent variables in the Cox regression analysis. The overall test of the model was statistically significant (*p* = 0.005). As shown in Table 3, third ventricular width was an independent predictor of cognitive impairment in PD patients (*p* = 0.011). Compared with the PD patients with a third ventricular width <4.75 mm, those with a third ventricular width ≥4.75 mm at baseline had a 7.975 times higher risk of developing cognitive impairment [hazard ratio (HR) =7.975, 95% CI 1.609, 39.532, *p* = 0.011].

**TABLE 3:** Cox regression analysis of third ventricular width as a predictor of cognitive impairment in PD patients.

	MMSE scoring criteria		*p*
	Third ventricular width <4.75 mm	Third ventricular width ≥4.75 mm	
Sample *n*	57	23	
New onset cognitive impairment *n* (%)	2.0 (3.5)	6.0 (26.1)	
Model	Ref	7.975(1.609, 39.532)	0.011*

Abbreviation: MMSE, Mini‐Mental State Examination.

* *p* < 0.05, which was statistically significant.

## 4 DISCUSSION

The primary objective of our study was to assess the value of TCS as a diagnostic tool in PD. Our findings revealed that older age and later onset age were associated with both larger third ventricular width and poorer cognitive function, indicating that age and onset age had an important impact on both cognition and third ventricular width. We further discovered that PD patients with cognitive impairment had a larger third ventricular width compared with those without cognitive impairment, suggesting third ventricular width as a potential diagnostic tool for PD with cognitive impairment. Similar observations have been reported in a TCS study examining physiological aging and dementia, which also noted an increase in the third ventricular width.^9^ The correlation between a larger third ventricular width and poorer cognition has also been confirmed in both AD and LBD through brain MRI.^8^ Furthermore, third ventricular width enlargement as a reproducible and clinically relevant markers of brain atrophy has been demonstrated by magnetic resonance.^7^ As a 2‐year follow‐up study reported, brain atrophy is a structural imaging marker of cognitive decline.^19^ Therefore, we propose that third ventricular width measured by TCS could serve as an imaging biomarker for cognitive impairment in PD patients.

In this study, third ventricular width identified cognitive impairment in PD patients with 62.7% sensitivity and approximately 75.6% specificity. While Aβ42/NG ratio in cerebrospinal fluid (CSF) as a screening tool have higher sensitivity (92%),^20^ it also come with drawbacks such as high costs, invasiveness, and difficulty in widespread adoption. In 2020, a cohort study showed that the optimal cutoff point for third ventricular width in the diagnosis of dementia was 4.65 mm in healthy Spanish individuals, which closely matches that (4.75 mm) in our study.^21^ Furthermore, our follow‐up study indicated that not cognitively impaired PD patients with a third ventricular width equal to or greater than 4.75 mm at baseline were independently associated with the diagnosis of cognitive impairment with an odds ratio of 7.975 [1.609–39.532]. This also aligns with the 7‐year follow‐up study based on healthy Spanish individuals, which similarly reported that a third ventricular width diameter equal to or greater than 4.65 mm at baseline was independently associated with the diagnosis of MCI/dementia with an odds ratio of 13.44 [3.59–50.37].^21^ These findings, along with previous report suggesting that ventricle enlargement and hippocampal atrophy occurred in the early course of PD,^22^ and several studies demonstrating that hippocampal atrophy was associated with cognitive decline,^23^, ^24^, ^25^ reinforce our study's conclusion.

The MMSE consists of five dimensions: visuospatial and executive functioning, language abilities, short‐term memory/delayed recall, attention, and orientation. In our exploration of the correlations between subdomains of cognitive function and third ventricular width, we discovered that third ventricular width was only negatively correlated with the memory subdomain. This finding is consistent with prior research indicating that ventricle volume measured by MRI significantly correlates with the memory domain score.^26^ In clinical manifestations, researchers have also found that the decrease in cognitive function of PD patients is primarily manifested as a decline in memory compared with cognitive function in the same age group of non‐PD patients.^1^ It is also noteworthy that hippocampal atrophy occurs early in the course of PD.^27^ Moreover, memory coding and storage function impairment is a primary characteristic in PD patients with early cognitive decline.^28^ This might explain the correlation between the ventricular width and the memory subdomain.

Previous study has identified an association between third ventricular width by TCS and disability status, and progression in multiple sclerosis patients has been observed.^29^ When we further investigate the correlation of third ventricular width with disease characteristics in PD, we found that there was no significant association between third ventricular width and the severity of PD (MDS‐UPDRS III, H&Y). Our findings matched Behnke's study results.^10^ Additionally, Behnke reported no significant difference in third ventricular width between men and women. However, we did observed a significant difference in third ventricular width between men and women, which was consistent with a longitudinal study on 500 subjects.^9^ This can be attributed to the known fact of men generally having larger brain volumes,^30^ which validates the TCS data.

Our study confirmed that third ventricular width was associated with the cognitive performance of PD patients, and a measure equal to or greater than 4.75 mm was a risk marker for the progression of cognitive impairment in Chinese PD patients. Nonetheless, this study is not without limitations. First, while the MMSE scale used in our study has been widely applied to assess cognitive performance in PD,^15^, ^31^, ^32^ it can evaluate only the current cognitive state but not the cognitive situation before disease onset. Second, the follow‐up period in our study was approximately 2 years, a duration insufficient for thoroughly observing the dynamic changes in cognitive function in PD patients. Third, the sample size of our study was relatively small. Finally, we did not collect and analyze heathy controls in our study. Despite these limitations, sonography‐based measurement of third ventricular width offers several benefits. It provides an image resolution of deep brain structures comparable to that of MRI.^33^ Also, TCS is characterized by a short examination time, reproducible results, and it is simple, inexpensive, noninvasive, and easy to acquire. Importantly, our study included follow‐up data.

In conclusion, we have demonstrated that third ventricular width based on TCS emerged as an independent predictor of developing cognitive impairment among PD patients. The findings could prove beneficial for clinicians in identifying the progression of cognitive impairment in PD, thereby enabling the prevention and delay of the onset of cognitive impairment.

## AUTHOR CONTRIBUTIONS

HG and ZX conceptualized the study design. HG, JL, QY, and ZM collected demographic and clinical data. AT performed transcranial sonography. HG, YQ, and SC undertook the data analysis. HG, SC, and ZX interpreted the results. HG wrote the manuscript. All authors provided feedback for revision and read and approved the final report.

## CONFLICT OF INTEREST STATEMENT

The authors have no conflicts of interest to declare.

## Supporting information


Data S1:

## Data Availability

The data that support the findings of this study are available from the corresponding author upon reasonable request.
