# Supplementary material for: Third ventricular width by transcranial sonography is associated with cognitive impairment in Parkinson's disease
Source: CNS Neurosci Ther. 2023 Jul 13;30(2):e14360. doi: 10.1111/cns.14360 (PMC10848047; doi:10.1111/cns.14360)
Supplement: Supplementary file 1 — Data S1: [file CNS-30-e14360-s001.docx]

**Supplemental Online Conten**t

**Supplementary Table 1|** Demographic and clinical data

| Variables | PD patients (n=174) |
| --- | --- |
| Female (n) | 56 (32.2) |
| Age, (y) | 60.00 (51.00, 66.00) |
| Onset age, (y) | 55.00 (47.00, 61.00) |
| Disease duration, (y) | 4.00 (2.00, 6.00) |
| Education level, (y) |  |
| Illiteracy (0) | 7 (4.0) |
| Primary (0-6) | 42 (24.1) |
| Secondary (6-12) | 102 (58.6) |
| University and above (>12) | 23 (13.2) |
| MMSE score | 26.00 (23.00, 28.00) |
| Visuospatial and executive functioning (0-6) | 5.50 (4.00, 6.00) |
| Language abilities (0-3) | 3.00 (2.00, 3.00) |
| Short-term memory/delayed recall (0-6) | 5.00 (5.00, 6.00) |
| Attention (0-5) | 4.50 (3.00, 5.00) |
| Orientation (0-10) | 10.00 (9.00, 10.00) |
| Hoehn-Yahr Stage | 2.00 (1.0, 3.0) |
| 1 | 50 (28.7) |
| 1.5 or 2 | 61 (35.1) |
| 2.5 or 3 | 49 (28.2) |
| 4 | 10 (5.7) |
| 5 | 4 (2.3) |
| MDS-UPDRS score |  |
| Part I | 9.00 (5.00, 13.00) |
| Part II | 8.00 (4.00, 13.00) |
| Part IIII | 26.00 (16.00, 41.00) |
| Third ventricular width (mm) | 4.40 (3.20, 5.60) |

Dates are presented as n (%) or median (interquartile range).

**Supplementary Table 2|** The demographic and clinical data of different sex in PD patients

|  | Male  n=118 | Female  n=56 | *P* |
| --- | --- | --- | --- |
| Age, (y) | 61.00 (52.00, 67.00) | 57.50 (50.30, 65.75) | 0.215^a^ |
| Onset age, (y) | 55.00 (47.00, 61.25) | 52.00 (48.00, 60.00) | -0.347^a^ |
| Disease duration, (y) | 4.00 (2.00, 6.00) | 5.00 (2.00, 7.00) | 0.633^a^ |
| Education level, (y) |  |  | 0.055^b^ |
| Illiteracy (0) | 3 (2.5) | 4 (7.2) |  |
| Primary (0-6) | 23 (19.5) | 19 (33.9) |  |
| Secondary (6-12) | 74 (62.7) | 28 (50.0) |  |
| University and above (>12) | 18 (15.3) | 5 (8.9) |  |
| Third ventricular width (mm) | 4.60 (3.50, 6.20) | 3.80 (2.80, 4.60) | 0.001^a*^ |
| MMSE score | 26.00 (23.00, 28.00) | 25.00 (22.00, 28.00) | 0.710^a^ |

Dates are presented as n (%) or median (interquartile range). ^a^ Based on Mann–Whitney U tests. ^b^ Based on chi-squared test. ^*^ means *P* < 0.05, which was statistically significant.

**Supplementary Table 3|** Correlation between cognitive function and disease characteristics in PD patients

|  |  | Spearman correlation analysis | | |
| --- | --- | --- | --- | --- |
|  |  | R | *P* | |
| Age, (y) |  | -0.301 | <0.001^*^ |  |
| Onset age, (y) |  | -0.236 | <0.001^*^ |  |
| Duration, (y) |  | -0.115 | 0.134 |  |
| MDS-UPDRSI score |  | -0.234 | 0.002^*^ |  |
| MDS-UPDRSII score |  | -0.111 | 0.145 |  |
| MDS-UPDRSIII score |  | -0.045 | 0.553 |  |
| 3Third ventricular Width (mm) |  | -0.327 | <0.001^*^ |  |
| Hoehn-Yahr stage |  | -0.070 | 0.357 |  |

The correlation coefficient r is defined as very weak (r = 0.00 ~ 0.29), low degree (r = 0.30 ~ 0.49), medium (r = 0.50 ~ 0.79) and strong (r = 0.80 ~ 1.00). ^*^ means *P* < 0.05, which was statistically significant.

**Supplementary Table 4|** Correlation between third ventricular width and clinical characteristics in PD patients

|  | Spearman correlation analysis | |  | Partial correlation | |
| --- | --- | --- | --- | --- | --- |
|  | R | *P* |  | R | *P* |
| Age, (y) | 0.582 | <0.001^*^ |  |  |  |
| Onset age, (y) | 0.541 | <0.001^*^ |  |  |  |
| Duration, (y) | 0.083 | 0.282 |  |  |  |
| Hoehn-Yahr stage | 0.049 | 0.518 |  |  |  |
| MDS-UPDRS score |  |  |  |  |  |
| Part I | 0.127 | 0.095 |  |  |  |
| Part II | 0.128 | 0.090 |  |  |  |
| Part III | 0.102 | 0.179 |  |  |  |
| MMSE score | -0.327 | <0.001^*^ |  | -0.205 | 0.008^*^ |
| Visuospatial and executive functioning (0-6) | -0.277 | <0.001^*^ |  | -0.118 | 0.129 |
| Language abilities (0-3) | -0.047 | 0.538 |  | 0.012 | 0.874 |
| Short-term memory/delayed recall (0-6) | -0.303 | <0.001^*^ |  | -0.262 | 0.001^*^ |
| Attention (0-5) | -0.214 | 0.015^*^ |  | -0.181 | 0.046^*^ |
| Orientation (0-10) | -0.185 | 0.015^*^ |  | -0.069 | 0.372 |

Partial correlation analysis: corrected for age and age of onset at the same time. The correlation coefficient r was defined as very weak (r = 0.00 ~ 0.29), low degree (r = 0.30 ~ 0.49), medium (r = 0.50 ~ 0.79) and strong (r = 0.80 ~ 1.00). ^*^ means *P* < 0.05, which was statistically significant.
